# Therapeutic emails

**DOI:** 10.1186/1747-597X-2-7

**Published:** 2007-02-16

**Authors:** Farrokh Alemi, Mary R Haack, Susanna Nemes, Renita Aughburns, Jennifer Sinkule, Duncan Neuhauser

**Affiliations:** 1 Department of Health Administration and Policy, College of Health and Human Services, George Mason University, Northeast Module, 4400 University Drive, MS 1J3, Fairfax, VA, 22030, USA; 2 Chair and Professor, Department of Family and Community Health, School of Nursing, 655 W. Lombard St., Room 616, Baltimore, MD 21201, USA; 3 Associate Professor, University of Maryland, School of Nursing, 655 W. Lombard St., Baltimore, MD 21201, USA; 4 NYCHA Classic Center Director, 555 Mt. Prospect Avenue, #17H, Newark, NJ 07104, USA; 5 Department of Psychology, David King Hall, Rm. 2003, George Mason University, 4400 University Drive, MS 3F5, Fairfax, VA 22030, USA; 6 Department of Epidemiology and Biostatistics, Medical School, Case Western Reserve University, 10900 Euclid Ave., Cleveland, OH 44106, USA

## Abstract

**Background:**

In this paper, we show how counselors and psychologists can use emails for online management of substance abusers, including the anatomy and content of emails that clinicians should send substance abusers. Some investigators have attempted to determine if providing mental health services online is an efficacious delivery of treatment. The question of efficacy is an empirical issue that cannot be settled unless we are explicitly clear about the content and nature of online treatment. We believe that it is not the communications via internet that matters, but the content of these communications. The purpose of this paper is to provide the content of our online counseling services so others can duplicate the work and investigate its efficacy.

**Results:**

We have managed nearly 300 clients online for recovery from substance abuse. Treatment included individual counseling (motivational interviewing, cognitive-behavior therapy, relapse prevention assignments), participation in an electronic support group and the development of a recovery team. Our findings of success with these interventions are reported elsewhere. Our experience has led to development of a protocol of care that is described more fully in this paper. This protocol is based on stages of change and relapse prevention theories and follows a Motivational Interviewing method of counseling.

**Conclusion:**

The use of electronic media in providing mental health treatment remains controversial due to concerns about confidentiality, security and legal considerations. More research is needed to validate and generalize the use of online treatment for mental health problems. If researchers have to build on each others work, it is paramount that we share our protocols of care, as we have done in this paper.

## Background

Surveys show that an overwhelming numbers of clients wish to contact their clinicians by email [1,2]. Despite the client's wishes, very few clinicians maintain email contact with their clients [3,4]. Providers' resistance to the use of email may be due to lack of training, the need to modify processes of care or misperceptions about the effectiveness of emails in maintaining a therapeutic relationship. Many investigators have attempted to determine if providing mental health services online is an efficacious delivery of treatment. As the internet is simply a form of media, it is the content of this media which will determine its effects. Therefore, it is important to understand what content is necessary to achieve desired outcomes for mental health services provided online. This information may also inform clinical policy, and address skepticism and negative attitudes toward the use of email in a therapeutic setting.

A number of investigators have examined the impact of online interventions on behavior change [5-12]. Interventions that include role-playing [11], electronic social support [11-13], and tailored educational messages [11,12,14,15], lower cost of care and improve health of clients. For example, a study at the University of Wisconsin provided 200 HIV clients with computer services, including an electronic support group, where clients could post written messages for each other. Clients were randomly assigned to control and experimental groups. Only the experimental group had access to the computer services. Investigators found that the experimental clients were more likely to report higher quality of life in several dimensions including social support and cognitive functioning. They also had fewer office visit (dentists, primary provider and alternative care providers), shorter primary care visits, fewer hospital admissions and shorter hospital stays. In summary, experimental clients had better mental health and lower total health care cost than the control clients [16]. The study by Gustafson and colleagues [16], and other studies mentioned earlier, has established the efficacy of online services in bringing about behavior change.

If online services are effective in bringing about behavior change, it is natural to extend these services to treating clients online. However, the suggestion that the internet may be a useful tool in providing psychotherapy services raises several concerns. The American Psychological Association (APA) Ethics Code [17] which presents guidelines for the provision of mental health services, cites a primary obligation of clinicians as taking precaution to protect confidential client information, and federal law now requires that healthcare providers to protect private client information. Client information, when transmitted electronically, is vulnerable to security and privacy violations. There is concern that transmitting private client information online will lead to litigation for privacy violations.

Recupero [18] reviews several legal dangers inherent in the use of email between clinicians and clients. The author points out that while many patients prefer to communicate with clinicians via email, this form of communication brings with it specific legal concerns. First, unlike telephone communication, email creates a permanent written record of what a client said, but leaves the exact meaning open to interpretation. For example, clinicians are under a duty to inform should they become aware that their client represents a danger to themselves or others. If a client verbalizes fantasies of harm to the clinician simply for the therapeutic effect of doing so, but without intention to act, a court could interpret what was said as an indication of intent to harm, leaving both the clinician and the client liable. Second, there is always some lag time between when a patient sends an email and when a clinician responds, which could be a liability if the client attempts to contact the clinician via email while in a crisis situation. Third, there is a chance of accidental misdirection of an email or interception by third parties, such as when several family members share an email account. Finally, email is not able to convey all aspects that are important in the communication between a clinician and a client, such as tone of voice, gestures, facial expressions, and the relationship between visual and auditory content.

Despite these concerns, both the American Psychological Association (APA) [17] and the American Medical Association (AMA) [19] condone the use of email and other electronic media for clinician-client communication, and offer several guidelines to ensure that it is used safely by clinicians and clients. Both the APA and the AMA state that clients must be informed of the risks to privacy inherent in transmitting private information online, as well as the security measures which most current servers now have in place to protect sensitive information (e.g. password protection for electronic documents, secure website or messaging service, encryption). They also suggest that clinicians obtain informed consent or a release of information from clients, giving clinicians' permission to communicate with them via email or other electronic media regarding personal medical information. This document should also clearly specify the types of transactions which are authorized to occur online. For example, as Recupero [18] suggests, the client should be informed that email should not be used in crisis situations, and should be provided with appropriate means of communication in these instances. APA and AMA also suggest that the clinician retain paper copies of electronic transactions with clients and store them in the medical record. Any emails which are unclear in meaning, or which could be interpreted in various ways should be clarified with the client, and record should be made of such clarification. Such precautions can make the use of electronic media in the provision of healthcare services possible.

Despite the potential of electronic media for providing psychotherapy, few investigators have used computers for treatment. Recently, two groups of investigators in the United States and England have focused on eliminating the need for a clinician and having the computer treat clients. Osgood-Hynes and colleagues have shown that clients can use a computer to effectively treat their own mild depression [20]. Griest and colleagues have shown that computers can treat obsessive-compulsive disorders [21]. In these two studies, computers organized the care, interacted with clients and monitored their progress. Most providers do not want computers to replace them but to assist them in interacting with their clients. This paper focuses on how email contacts can enhance the bond between the clinician and the client. In the proposed intervention, the computer is not the treatment but the medium through which treatment is delivered. The clinician's interaction with the client remains the core activity.

Computer facilitated counseling has been tried in the past. Friedman and colleagues showed that computer facilitated counseling can help hypertensive clients reduce their blood pressure [22]. But it has not been tried in substance abuse; with a few exceptions. In the last decade, we have conducted a number of linked studies on the potential impact of various components of online treatment on recovering substance abuse clients. We describe this research below. We found that repeated use of online services was positively correlated with retention in substance abuse treatment [23].

In the first study [23], 82 cocaine-using pregnant clients had access to online services through their telephone line. In addition, both groups received existing treatment for substance abuse, case management and prenatal and pediatric healthcare. Professionals in charge of the participants' care used the system to encourage and monitor treatment, but no standard protocal existed for what information needed to be communicated. This was left to the opinion of the professionals consulting with participants. Results showed that of those who had access to online services, 35% used the system more than 3 times a week. Eighty two percent of clients who used the system more than 3 times a week participated in treatment in contrast to 55% of the clients who used the system less than 3 times a week or not at all. More frequent users were 1.5 times more likely to be in community-based treatment. Similarly, clients who used online services 3 times a week were 1.7 times more likely to participate in self-care, such as Narcotics Anonymous. Thus, more online use was associated with more treatment and more self-care, two factors that predict long term effective substance abuse treatment. While not measured explicity, it must also be noted that willingness to receive services via computer also likely contributed to receiving more treatment.

In a second study [24], the impact of electronic self-help groups on 53 recovering pregnant women was examined. Subjects were recruited from previously randomly assigned groups. One group had access to online services and was provided with an online support group. The other group was provided with only an in-person support group. Both groups were monitored and facilitated by a counselor, who provided discussion topics for the groups including stress; violence in my community; patient-doctor relationships; emergency room treatment; parenting; housing issues; welfare reform; being black, female and sober; child abuse; welfare fraud and entrapment. Biweekly participation rates for the online support group over four months ranged from 54% to 79%; in contrast the participation rate in the face-to-face group ranged from 0% to 20%. Clearly, though not surprising, clients found the online participation to be easier and more convenient.

What surprised us were the clients' comments online and the impact of these activities on quality of care. We analyzed the comments clients made online. Among the comments, 67.3% were intended to provide emotional support to other clients. The remaining comments were task oriented (e.g. announcing a community program). None of the comments involved flaming or overt disagreements. The more clients used online services the more they felt a sense of solidarity with each other, as measured by standardized measures of social norms. In other words, online support groups created the same types of emotional engagements that one expects in face-to-face meetings. Online groups behaved as if they were face-to-face. Rotondi and colleagues [13] also found this to be the case in an online support group provided to women who were primary caregivers to their husbands who had a traumatic brain injury.

A further surprise came from the impact of these online groups on recovery and cost of care. We do not have data regarding the cost of the delivery of online counseling compared to face to face counseling. However, our experience shows that the cost might be significantly lower than face to face therapy. There was no statistically significant difference in recovery rates but there was a significant drop in utilization of health services. The control group in our second study of pregnant women suffering from substance abuse described above had 42% to 164% more visits than the experimental group across office and clinic visits for mental and physical health. Furthermore, by delivering services online, more patients can be served by a single clinician than would be possible in face to face settings. For example, in one project on online education, 240 patients were served by one counselor [14]. Our results and others such as the example cited suggests that cost of delivery of online services might be significantly lower than face to face treatment. Additional data is needed to further establish the relative cost of online and in-person therapy.

These studies raised the expectation that online services could complement substance abuse treatment, and the authors were interested in determining if the successful efforts described above would generalize to the use of email in on-line treatment. We were asked to provide supplemental online services to Target City clients in Cleveland. Clients seeking treatment were assigned to different outpatient treatment programs through the Cleveland Target City central intake unit. At the time of assignment to an outpatient setting, these clients were also invited to participate in online services. Surprisingly 87% of clients participated regularly in online services. But only 30% of the same clients showed up for their first outpatient appointment. Clearly, 50% of the clients received only online treatment. Although we were tasked to enhance treatment, for at least 50% of the subjects we were the only care they received.^15 ^Note that these data show additional participation in online treatment and not improved outcomes. Because of additional participation, we quickly had to develop and put into use content that covered the entire treatment and therefore could replace as opposed to augment treatment. The nature of the treatment provided online will be discussed in further detail below, but it is pertinent to point out that despite our successes with online treatment, other providers were not obtaining the same results. We began to see the need for more documentation of what we were doing online so that others could replicate our model. In addition, over the years, we have come to see the fallacy of evaluating the impact of online services: It is not the availability of the online service that creates the impact; it is the content of what is said and done online that is important. Because of concern with content, scientists who have tried to replicate or improve upon our studies have repeatedly asked us for a blueprint. This paper provides a detailed description of how email can be used in treatment of substance abuse.

## Results

### Anatomy of an email

Every email has distinct components. Some components (e.g. email address) are necessary to the function of an email; other components are unique to the type of emails we send to our clients and thus can be thought of as a necessary component of the therapy. The components of emails we use are the following:

1. The clients alias (de-identified for the protection of the clients)

2. The counselor's name and title

3. The counselor's work email

4. Date email sent and date email opened

5. Time it takes for the addressee to open emails

This information is calculated by the computer in order to monitor whether emails remain a viable method of communication.

6. Greetings, including salutation

Typically the email should start with "Dear Alias", followed by "How are you? I hope all is well."

7. Statement of the issue

This is the core message of the email. We encourage counselors to send only one message per email.

8. Optional example so that the issue is well understood

The counselor may give an example from his/her own life or other clients' experiences with the issue.

9. Leading question

All emails need to end with a leading question that verifies the client's understanding of the issues raised by the email and propels the client to respond to the email.

10. Signature and title of counselor

11. Statement of confidentiality of email communications

Most of the elements mentioned above are present in email programs. The key exception is ending of emails in leading questions. This is a key aspect of all Motivational Interviews. It is an important strategy for increasing the client's insight to the problem.

### Content and progression of emails

The nature of the interaction between the clinician and the client depends on the recovery process of the client and cannot be specified a priori. All individuals are distinctly different and the course of the counseling is uniquely different for each person involved. But there are distinct stages that clients go through, and at least in the early phases of these stages the content of the communication is relatively standardized. This section provides an overview of the ten stages in recovery and the content of emails in these stages. These steps describe progression from thinking about change to succeeding at it; a progression from individual to group action. While some clients may not go through all of these steps, or may skip steps in their progress, it is our experience that many do proceed sequentially through the change process. Furthermore, when each one of these steps is tied to specific rewards and sanctions, clients are more likely to progress through them. Over time and across a large number of people, we have identified several characteristics of treatment at each stage which appear to promote success in utilizing online treatment for substance abuse. These characteristics will also be discussed in this section. A case example of the progression of treatment described in this section can be found in the appendix.

#### 1. Establish contact

In this stage, client and the clinician introduce themselves and verify that they can receive and send emails. For example:

"Hello I am (short description of the clinician's background). I want you to know that our discussions are confidential and will not be reported elsewhere without your permission. Some exceptions to this are if a court orders us to share our records or if our discussion reveals an activity that by law I am required to report. Keep in mind that emails can be read on route to me by others. To keep your privacy, please do not put your name or phone number in emails. I have your name and phone number and you do not need to send it to me. Before we get started I wanted to know more about you. Could you tell me how old are you, who you are, what you do, and any thing else that you think will help me understand where you come from. Would you introduce yourself?"

This step is most successful when the client has agreed to and does participate in treatment online. Clients who receive and respond to messages quickly (24 to 48 hours) are successful in this phase of treatment.

#### 2. Assessment

The clinician asks why the client is seeking online counseling in order to help the client articulate in his/her own words their drug dependence. Often clients are referred to online counseling after a specific intake interview. The clinician could refer to these intake interviews and ask:

"This is xxx. I want to start by asking you why you are here. I have seen your responses to the intake interview. But I want to hear from you and in your own words. For example, some people come to online counseling because they have to; others come to talk to me because they want to accomplish a change in their life. Can you tell me why you are here?"

The aim of these early conversations is to make the client and not the clinician use words such as "alcohol or drug use." Then the clinician follows by asking about the positive feelings the client gets from his/her drug use:

"This is xxx. I want to ask you about some of the good things that you think you get from your drug or alcohol use. What do you get from it? In the past when I have asked this question from other clients they have told me what they enjoy when they use. Some have said they enjoy being trouble free, others have said they enjoy their friends. What is it that you enjoy when you use? What do you get out of it?"

Success in this phase of treatment is dependent upon the client gaining insight into why he/she is ambivalent about changing substance use behavior.

#### 3. Identify consequences of substance use

The clinician starts without focusing on the drug or alcohol use.

"This is xxx. I want you to tell me some of the things that might be a problem or concern for you now. If you ask me this question, I would list money and relationships as my top concerns. But I want you take an inventory of your life and tell me what is not going well. Could you list the issues that are of most concern to you recently?"

If the client is not sure what is meant, examples are given:

"This is xxx. It was good to see your last email. You were not sure what I was asking about earlier. Maybe an example can help. Some people are concerned about their health, relationships or finances. Do you have any concerns such as these?"

When broad concerns are raised, the clinician asks for more specific examples:

"This is xxx. You said you were concerned about .... Can you give me a specific example, a case where this caused you problems or difficulty?"

The clinician provides confirmation, brings forward statements made about how drug use is enjoyed and seeks explanations for why certain events have occurred:

"This is xxx. Earlier you had said that you enjoy your habit because ..... Now you are saying that you are concerned about ... Lets continue with this line of thinking. I want to get to understand your concerns more fully. What has contributed to these problems? If you ask me about my life and what has led to my current problems, I can list for you a number of people who have failed to help me. But in the end, I have to admit that there are a number of things that I have done that have made my life much harder. What have you done that has contributed to your problems?"

The clinician does not explicitly point to the relationship between the drug use and the concerns raised by the client, but if the client mentions such relationships, then they are reinforced by the clinician. If by now concerns are not linked to the drug use, the conversation is changed to a future focus.

"This is xxx. We discussed some of your present concerns or problems. Let me ask you about the future. If you were to continue your current lifestyle, what concerns might you have about the future?"

When drug use is acknowledged, the clinician spends more time on the link between drug use and life problems.

"You say that drug use has contributed to your problems. I want you to send to me an email about things that happen to you that you did not like when you used drugs. In a month or so from now, when you are doing better, I will send the message back to you so that you can see where you came from. As we go through recovery, sometimes we forget why we wanted to stop using drugs and hearing yourself again is a good reminder. So please reply to this message and tell me in as much detail as possible how your drug use creates problems for you. Could you give me the story of the worst experience you have had because of drug use?"

In the end the clinician summarizes the concerns and directs the client forward in treatment with a message such as:

"This is xxx with a summary of what we discussed so far and a request. Let me see if I can pull together our discussion to date. You have several concerns. On the one hand, you are concerned about (give the details of a specific event that raised the concern). As far as the future goes, you are worried that some of these things may get worse.... On the other hand, you enjoy your habit. You mentioned that you like ... I can see from your responses that you have thought these issues. But most important, you mentioned that your habit contributes to your problem. In your words, "...". It is great finding a person who can think through both the positive and the negative parts of your habit. Most people muddle through unaware of their lives. You are different. I am glad that you are aware of these and how they affect your life.... We have passed an important milestone in your treatment. Are you ready to proceed to the next stage?"

It is essential for the client to thoroughly explore and understand the consequences of substance use in order to motivate positive change.

#### 4 Develop a plan for recovery

The clinician summarizes the discussion to date and initiates a plan for recovery.

"Hi this is xxx again. In the past weeks, we have been talking about the consequences of your drug and/or alcohol use. On the one hand you feel that there are some things that you get out of using drugs, things that work for you. But there are also many negative consequences and you are concerned. Let's move on and see if we can come up with a specific plan that can help you change your drug use. I have mailed to your home address a brochure that I need you to sign and return. It is called "Contract for Change" and it highlights the activities that we would do together to change your drug use. I should tell you that we can succeed if that is what you want to do. In the end, it all depends on what you want to accomplish. I cannot decide for you. I only can act as a coach and cheer you on when you succeed and show you other ways when you fail. Would you let me know when you have received the brochure?"

At a later point, the counselor asks for how the client plans to quit drug use:

"Hi this is xxx again. You sound like you are ready to stop using. When people want to change their behavior, they need to set a date, engage in numerous preparation activities, have public rituals marking the change and create ways of keeping up with their resolutions. I need to hear from you regarding what are your plans. When do you want to stop or if you have stopped already when you plan to do so that you can keep your resolution?"

This step also marks the beginning of the individual's attempt to stop his/her drug and/or alcohol use.

"Hi again. This is xxx. I wonder how you are doing and if you have been successful in changing your drug use. Tell me how you have been trying to change, where has there been set backs and what you intend to do."

Often clients' plans are not concrete. Clinicians emails should solicit specifics from the client regarding who, what, where and when they will take action. It also helps to ask for the actions the client might take immediately, in an hour and in a few days.

"This is xxx. I can see you have thought through the actions you want to take. Can you help me by telling me what you will do immediately after this email, what will you do in an hour or so, what will you do tomorrow and what will you do this weekend so that you will stop drug use Be specific. Tell me how you have been trying to change, where has there been set backs and what you intend to do at specific times this week."

Clients who develop a clear and specific plan about actions they will take to initiate change are most likely to successfully negotiate this phase of recovery. This plan should include practical strategies for coping with set backs and strategies for promoting success.

#### 5 Admit to substance abuse and mobilize support for change

The clinician would next follow up about the client's plan to change substance use behavior via the following message:

Hello, this is xxx. I was wondering how you are and also wanted to remind you what we agreed upon earlier in the Contract for Change. You agreed that you will admit to your friends and family members that you are powerless over your drug addiction and that it has become unmanageable. Have you done so? Who have you talked to about your situation?"

In response to the message, the clinician will address the concerns raised and then also end with the following message:

"This is xxx again with an idea. Why don't you tell the people online about your intention to change your drug use? Select the discussion group. When you go there tell them your first name and admit to them that your drug use has become unmanageable. You will find that they also need your help."

Later, the clinician will ask for a summary of reactions the client has received from others:

"This is xxx. I hope that by now you have admitted to people you know and the people online that you need their help. If you have done so, I like to hear from you what reactions were and how you felt."

The clinician helps the client to engage others in the change he/she is contemplating:

"This is xxx. You are not alone in this. There are many people who can help you. Some are online and some are right there with you. You can get help from clinicians. But the most important people that can help you are family and friends who care for you and want you to stop using drugs. You need to sit down with them and get their help. Your recovery needs to be a team effort. There are a lot of things in your environment that need to be changed for you to succeed. You have been making a great personal effort to succeed but now it is time to engage others so that you can modify your environment and succeed even when your resolve fails. If you ask me who should be part of your team, I suggest those who are most affected by your drug use and with whom you share daily living activities. A brochure describing how they can help you is available through us. Could you put together the list of people who share daily living activities with you and who might participate in the recovery team? Who do you suggest should be on your team?"

The clinician follows up a few days later:

"This is xxx. I had asked you to put together a team that can help you change. You may wonder what this team should do. Let me summarize some key points of what will be expected from your team. The team's goal is to adjust daily routine activities so that is no longer possible or desirable to continue using drugs. For example, if I was trying to lose weight, an adjustment that I could come up would be to shop for healthier food or food that is less fattening. There are several ground rules for how the team members should interact. First, there should be regular meetings. Second, no one is to be blamed. If people fail to carry out the task ask yourself how could we reorganize activities so the people involved can keep up with their resolutions? Third, the team should look for solutions that involve all or most team members and not actions that can be carried out by single individuals. As I mentioned earlier, I can send you a brief guide to how you can work together. If you have succeeded in getting people to help, let me know how many people are involved so I can mail you the guide. If you are concerned about how the recovery team can be organized, what the team will do and who should be on it, email me your questions

A detailed guide to organization of recovery teams is available through the first author.^7^

Clients who are able to admit to substance abuse and the need for help to those who are close to them are successful in this phase of treatment.

#### 6 Identify problematic interpersonal relationships

The clinician leaves the following message:

"This is xxx again. One of the secrets for not using drugs is staying away from people who use drugs. You should think about all the people you have contact with and ask yourself which of them use drugs. Then you should make plans to see friends and family members who do not use drugs and avoid contact with those who do. Are you willing to work with me on this? If yes, please reply by making two lists of first names. In the first list record the first name of all the people you enjoy being with who don't use drugs. In the second list, record the name of the people who use drugs.

Conversation continues until the client arrives at specific plans for how to increase contact with some friends and avoid others. Here is a prompt that can direct the conversation to role playing how to refuse drugs.

"This is xxx. I am going to tell you the beginning of a story and I want you to complete the story. Suppose you are out having fun with your friends. They begin to use drugs and offer you some. You say "No." But there you are sitting and watching them have fun. Your mind tells you must leave but you know that you are going to feel miserable without your friends. Complete this story for me. Tell me what happens next and how you feel about it."

Next the client is probed for what they might do to feel good about refusing drugs.

"This is xxx. Saying "No" is an art. It is more than just saying you don't want something. In a moment I want you to practice by saying "No" to me. When you do I want you to keep in mind the following components of saying "No." When refusing drugs, the first thing you say is the word "No." Then you should follow this with instructions to the person not to ask you now or in the future again. Don't make statements like "Maybe later." Make sure that your expression and tone are clear. Then offer something positive that is incompatible with drug use. Make sure that you end your comments with a question that focuses your friend's attention on a different topic of conversation or activity. Let's see if you can do this. Suppose I am your friend and I am offering you drugs. Practice with me how you would say no. Say no to me and I will tell you if you followed my instructions about how to say no."

The client and the clinician may also switch roles with the client offering drugs and the clinician saying "No." If during the intake or other conversations it is clear that the person is living with a person who uses drugs or alcohol, the following message is sent:

"This is xxx. If you are living with someone who uses drugs or alcohol, there are several things you can try. You can ask them to change, help them get professional help, and refer them to treatment. You can sometimes ask them to move out if they do not change. It is hard, but you can also move out yourself. But if neither strategy is possible, consider how you can separate as much of your life from them as possible. Think about eating, sleeping, watching television, or socializing and think how you can do these things separately without the involvement of this person. This is a hard issue but sooner or later you have to face it. Let's do it now together. Please tell me what you are planning to do to reduce contact with people who still use drugs."

Four weeks later the clinician sends the following message:

"This is xxx. You had wanted to decrease your contact with xxx. Have you succeeded? What do you think can help you get there faster?"

It is imperative that the client take concrete steps to reduce contact with friends and/or family who continue to use alcohol and drugs for successful completion of this phase of treatment.

#### 7 Adjust daily routines through group action

The clinician needs to make efforts to help the client engage a social support network to help in the process of recovery. The following assumes that the contact with the support network is through the client. The clinician helps the client to go beyond immediate commitment to stopping the drug use and identifies other steps which should be taken to create a positive environment the promotes success. The clinician sends the following message:

"This is xxx. It takes more than willpower and discipline to stop using drugs. You need to create an environment that helps you succeed. How can you and the people who are helping you adjust daily living activities, like when you eat, when do you sleep, etc. so that you are less likely to relapse?"

If the client insists on solely relaying on his/her personal motivation to stop using, the clinician continues:

"I believe in you and know you can succeed. But I need your help in understanding your drug use. Think about the time, money, motivation, and means that go into your drug use. Then think about how you can plan your days so that there is no time for it, or that you don't have the means for it, or that you are motivated to do something else. I need you to think of some actions that you and people helping you can take that will change the environment."

If an action is suggested, then the clinician explores whether there is a consensus from others about this course of action.

"This is xxx. You suggested ... who else would this affect? Do you have their agreement? Have you discussed it with them? Given how much work needs to be done, you need to ask for their help. You would be more successful to define the problem with them in a way that highlights how it affects them and to search for a solution jointly and not individually. Take these steps: work on changing the environment together and in a few days I will ask about your progress."

After a few days:

"This is xxx again. What have you come up with? I had asked you to think of specific actions that would remove the motivation and means for your drug use. Can you tell me what you have come up with?

The clinician can also explore whether the client has carefully examined his/her routines and how they may interfere with the proposed activity.

"This is xxx. To accomplish what you want to do several other activities should be done first. For example, if I was trying to stop alcohol use, I would examine when I drink and see how it is related to various daily routines. If you are trying to... what other steps should you and others who are helping you take to increase the chances of success."

The messages are continued until a detailed plan that is small in scale is organized.

"This is xxx. We have been thinking together about how to change the environment. Are you willing to give it a try? Now that you have found several things that you can adjust in your daily routines, why don't we set a start date?"

A week later the clinician should follow up with the client:

"This is xxx. I was wondering how you are doing. Did you succeed in accomplishing your plans? What changes in routine activities did you make and what others you plan to make?"

Clients who are successful in this phase of recovery are those who have been able to form a recovery group made up of supportive family members and friends. Success is also promoted by regular meetings between the client and the recovery group.

#### 8 Create a sense of spirituality & community through group action

The following message is sent by the clinician to the clients:

"This is xxx. Many of us believe that a power greater than us can restore us to sanity. Some of us have decided to turn our will and life over to god, as we understand god. It is helpful sometimes to talk about these issues. Can I ask you what you believe in? Do you believe in a higher power and how is this higher power helping you cope?"

The clinician will query more about client's sense of community and belongingness:

"This is xxx. Can you describe to me the community of people you identify with? Are they the people you meet at church or organized religious activity? Do you feel a sense of belonging to a group?"

The clinician continues the conversation to explore the client's need for and ability to arrange for a community of like-minded people. If during these conversations it is clear that the client believes in a god or is positively oriented towards spirituality, the clinician also makes the following offer:

"I know your life is busy. I wonder if you would like to receive messages of prayer and spirituality on the system. If you do, send me a message back."

Clients who feel a sense of belonging to a community, such as a religious group, and who feel supported by the group are more successful in their attempt to stop substance abuse.

#### 9 Identify substitute routines through group action

The clinician sends the following message:

"This is xxx. Stopping drug use is easier when it is replaced with a fun and healthy social routine. Some examples are walking in the morning, attending recovery events and meetings, such as NA or AA, or helping others in the neighborhood. I know that there are many things that you are interested in. I want to spend the next few weeks thinking through what you like to do and how you can make that happen regularly. Let's start by deciding what is it that you could do that could replace your drug use. Think about when you typically crave drugs. Think about a social fun activity that you can do at those times. Please think about your interests and strengths and tell me what is it that you really would like to do."

If the interest is reasonable, the clinician follows with:

"This is xxx again. Yes that sounds like fun. How would you get started? Let's spend some time together thinking through how you can plan this so it will happen. What steps will you take now, tomorrow and this weekend so that you can succeed at your plans?

Clients typically plan by committing themselves to actions. The clinician works with the client to make sure that a system-wide action is contemplated. The clinician can also explore whether the client has carefully examined his/her routines and how they may interfere with the proposed activity.

"This is xxx. To accomplish what you want to do several other activities should be done first. For example, if I was trying to lose weight, I should shop for healthier and less fattening food. If you are trying to do... what other steps should you and others who are helping you take to make sure that the means and the environment for success is at hand."

The relationship among the various habits of the clients is further explored.

"This is xxx. I want you and others who are helping you to think through how daily routines affect what you are planning to do. By daily routines I mean anything that repeats on a regular basis. For example, waking up, washing, going to work, preparing food, socializing, exercising, watching TV, visiting friends, etc. I want all of you to discuss this and then reply to me about how routine activities may prevent you from accomplishing what you want to do. I also want you and others who are helping you to think aloud about how daily routines can be adjusted to promote what you want to do. Is there a small specific change in daily routines that will lead to the activity you are planning? The idea is to change routines so that your planned activity would happen without much more additional thought or effort. In short, you need to make the new activity happen automatically and as a consequence of other routines – without having to think about it. What do you think should be done?"

The messages are continued until a detailed plan that is small in scale is organized.

"Are you willing to give it a try? Now that you have found several things that you can adjust in your daily routines so that you can achieve your plans, let's set a start date.

A few days before the planned activity the clinician sends the following message:

"This is xxx again. I know that your big day is coming up. I want to tell you something that I have learned time after time. One must work hard at having fun. Think about it. You can devote as much time and money to this new fun activity as you used to devote to your drug use. You can read books about it, purchase equipment for it, dress up for it and do what you need to make it a success. I am sitting here thinking of you and wishing you much fun."

A week later the clinician follows up with the client:

"This is xxx. I am wondering how you are doing. Did you succeed in accomplishing your plans? What changes in routine activities would make you succeed even more next time?"

Success in this phase of treatment is promoted by family and friends of the client altering daily routines to make it difficult for the client to continue drug use. Success is also promoted by the client replacing drug use with other fun activities which do not have the negative consequences of substance use.

#### 10 Share success with others

The clinician starts the conversation with the following message:

"This is xxx. Every day counts. Like the uncertainty faced by cancer patients, there is no guarantee that you will not struggle with your disease in the future. Each day that you are free from drug use is a gift and each time you participate in treatment is an action preventing relapse. I want you to start counting the days free of drugs and telling your friends and family members how many days has it been. One way that is often useful is to display in a prominent place, for example on the refrigerator, a calendar in which you could mark the days free of drugs. For my records, are you counting? How many days has it been? Have you been displaying the number of days you have been drug-free?

The client is also encouraged to share their data on use with the group that is helping him/her. Sometimes, with the client's permission, the clinician can take the role of informing the group through online methods. If it has been more than 30 days, the clinician sends the following message:

"That is good news. You have made it this far. I have an idea. Why don't you go to the discussion group and tell them of your success? People who are just beginning are struggling with their drug use. It will help them if they hear from you that you have made it this far."

A week later the following message is sent:

"This is xxx. I know you have made a great deal of progress by getting this far. If you ever relapse, don't let yourself go all the way. As quickly as possible start again on the right track. Let me or xxx (a name obtained earlier through the contract) know. We can help. "

Clients who have publicly shared their success stories and who have engaged family and friends in evaluating progress are most successful in this phase of treatment.

#### 11 Address cycles of relapse

The clinician asks for reports of relapse and asks the client to articulate what can be learned from this.

"Hi this is xxx. When you are trying to change, from time to time, you relapse. The key is to bounce back and not to become disappointed. Each relapse is an opportunity for new set of actions, which would lead to a longer period of being free from your drug use. Your recent email said that you relapsed. It is difficult to think through the sequence of events leading to drug use. But let's start by you telling me when the drug use happened, who were you with, where were you at, how were you feeling and why you think it occurred."

Once the circumstances around relapse have been explored, then environmental changes are solicited:

"This is xxx. I can see what happened. What can you do so the relapse into drug use will not happen again. Obviously you can exert more effort and remain more committed. But this is not what I am asking for. I need you to think about your environment and tell me what in your environment can be changed so you will not relapse again."

If the client responds with the importance of remaining committed, the clinician tries to help the client to devise strategies to prevent similar situation.

"This is xxx. You said that you have learned to be more committed and I believe you. But this is not what I was looking for. You said ... Ok what else could you do? I mean, is there an adjustment to your daily living activities that will reduce the likelihood of these types of relapses?

The messages continue until the client arrives at a specific adjustment to daily living activities that can help reduce the likelihood of relapse. It is imperative that both the client, as well as the friends and family members in the recovery group, have examined failures and arrived at adjustments to daily living activities in order to increase the intervals between relapses.

#### 12 Make amends and offer help to others

The clinician sends the following message:

"This is xxx. You know that you have a drug addiction which is a disease. Having this disease is not an excuse for anything – not for missing work, messing up family, stealing or breaking people's faith and trust. I think you have come to a point that you can come clean with the people that have been affected by your drug use. Are you ready to make a list of persons who have been harmed by your drug use and willingly make amends to them? Think about it, while it seems you are doing it for them you are really do it for yourself. Is there someone that you want to approach and tell them that you are sorry for what has happened?

The conversation is continued until the client selects a person, role-plays what the client will say, and plans a specific activity. After 90 days of sobriety, the clinician will ask the client to help others with the following message:

"This is xxx. You have come a long way. Having had your success and spiritual awakening, it is time that you carry the message to others. If you come into contact with a person who uses drugs, I want you to ask them if s/he is willing to help you by listening to your story. Then him/her what happened to you and how you were when you were using drugs and how you stopped. If after listening to you, s/he agrees to seek help, ask them to send us an email. Help them send the email if s/he is afraid to do it alone."

A week later the following message is sent:

"This is xxx. Are you still free from drugs? If so, I want you to go to the discussion section and under the topic of stories type your success story. I want you to tell your story; from the beginning to the end. Keep in mind that your identity is protected and that you should not use your name or phone number in any communications. Record your alias, tell how did you start using, what did you feel while using, and how did you succeed in stopping? When you are done, you should post your message so that others on the system can learn from you. I am really proud of your success to date."

Clients who help others recover from substance abuse increase their own chances of remaining sober.

## Discussion

Preliminary research has suggested that online interventions does impact participation in treatment and may impact behavior change. This paper has presented one approach to online counseling for substance abuse. We have also provided a case example in the appendix. The case highlights a number of issues in online counseling. In particular, it highlights the use of leading questions in getting clients to arrive at self-insight. It shows how clients come back after relapse to continue with their care. The case also highlights a number of potential problems. First, it shows that there is a risk that the counselor may fail to address important issues. In the case example, the counselor may have not actively addressed the client's anxiety – though it is not clear from the transcripts if the issue was addressed in phone conversations. Second, technical problems may be an issue. The case example highlights a series of technical problems in getting the client and the counselor to communicate with each other which hindered the course of treatment. Despite these setbacks, the case shows the start of a therapeutic relationship. It shows how online therapy can be used to engage clients, and how clients progress through a number of recovery stages. The case presented suffered a 16-day alcohol binge and was able to return to treatment, which serves as a useful example of how online treatment can succeed, even in the face of relapse.

## Methods

### Overview of online treatment

Our ideas for online services have been influenced by the Continuous Self-Improvement Theory [26] and recent work on situational determinants of relapse [27-33]. These studies emphasize that habits emerge from and can be challenged by altering the environment. Like these studies, our counselors focus on the relationship between daily routines and relapse to drug use. Lasting change occurs when the individual modifies daily routines and not just his/her own behavior. The counselor's role is to shift attention from client's personal effort to environmental changes that involve a number of people and that have more lasting impact on the behavior of the client. To this end, the online counselor helps the client organize face-to-face recovery teams that work with the client to change the environment. Our approach to online counseling also relies on Cognitive Behavior Therapy [34] in the sense that we emphasize a functional analysis of substance abuse, individualized training in situational determinants of relapse, and activities in between sessions directed toward relapse prevention. Our work has also been influenced by the work of Miller and Rollnick [35]. These authors have identified the use of "motivational" interviews to change addictive behavior. In this approach, the counselor does not tell the client what to do about his or her problems, but focuses on the client's own perception of the problems and what the client would like to do to attempt to change the situation. The two key components of these types of interviews are:

(1) Getting the client to think through the consequences of his/her actions and accept personal responsibility for those actions.

(2) Helping the client articulate why she/he is ambivalent about stopping substance abuse and to develop a commitment to change.

Motivational Interviewing is especially well suited for online delivery because it relies on dialogue between counselor and the client. Through these dialogues, counselors could be further reassured that their comments and questions are understood by the clients. At the same time, it is our experience that clients prefer to talk to rather than listen to the counselor. Because Miller and Rollnick's work emphasize directed questioning of clients in order to stimulate behavior change, their work is especially suitable for online delivery. Motivational interviewing has gained empirical support for use in treating substance abuse [37-40].

Our model of online counseling for substance abuse involves the maintenance of a longitudinal therapeutic relationship for 4 to 6 months. Both clinicians and clients must be aware that the focus of the activity is on long term change and that the impact cannot be observed for several months. During this period, the client is expected to be in near daily email contact with the clinician. Online treatment is more than frequent emails. In order to put the role of emails in online treatment in perspective, it is necessary to understand the various services available to the clients online. These services are as follows:

#### (1) Written contract for change

The contract lays out the specific plan for change. It also provides consent to steps that the clinician can take in case of client's relapse. These activities include (1) increasing online contact, (2) asking a significant other to talk with the client, (3) a telephone call from the clinician, (4) a clinic visit or other interventions as agreed upon.

#### (2) Electronic support groups

Clients use the electronic support groups to discuss recovery issues with others in recovery. Clients' comments are not censored, except when they involve illegal activities. All discussion groups have a sponsor that monitors the comments. For clients who share a specific characteristic or who have attained a specific level of progress, separate discussions are organized.

#### (3) Online motivational interviews

Motivational interviews are carried out through daily email contact with clients. Details of these emails are presented in a later section.

#### (4) Relapse prevention assignments

On a weekly basis, clients receive a survey of their environment. Clients' responses provide them and their counselor with insights into risks for relapse. If client is at increased risk, the counselor may take a number of steps including, (1) increased online contact, (2) asking a friend or a family member to spend time with the client, (3) arranging for face to face visit or (4) other agreed upon steps.

#### (5) Diary

On a daily basis clients are asked to report their drug use. When an occasional relapse occurs, clients are asked to specify the circumstances around it in order to help the client gain insight into how their environment can be modified to avoid future relapse. When the relapse is significant and suggests a return to drug use, the computer alerts the counselor who takes additional actions to help the client return to sobriety.

#### (6) Recovery teams

Members of the household, friends and others who share daily living routines with the clients meet once a week to discuss how these routines may be contributing to drug use. The members of the team flow chart the events that proceed relapse and identify daily activities that hinder drug use or maintain sobriety. The counselor has weekly contact with recovery team and helps set the agenda for these problem solving meetings. More details on the organization of recovery team are provided elsewhere [36].

In addition to these online components, there are two other elements important to delivery of online services. First, clients have access to face-to-face counselors if they wish to come in or if the counselor believes a visit is necessary. Second, clients are subject to random testing for substance abuse to verify their claims of no use.

The protocol provided here is a starting point for online counseling. These interactions trigger the conversation and are not the entire content of what transpires between the counselor and the patient. Sometimes patients do not progress from one stage to another. At first, clinician may continue interacting online until the patient comes to new self insight. If within 2 weeks it is clear that the patient is not progressing, the patient is called and interaction is continued on the phone. If by 4th week sufficient progress is not made, it is important to change the therapeutic modality. The patient should be asked to come in for a visit.

Many components of the intervention (e.g. recovery teams and peer to peer discussion groups) have not been described in this paper. Recovery teams have been described with more detail in a separate publication [18]. Content of peer to peer discussion groups is not set a-priori and therefore was not presented in this paper.

## Conclusion

We have described the details of our online interventions in the hope that researchers can test and improve these interventions. Our approach, as well as all thoughtful treatment programs, is constantly in revision. We continue to learn from the counselors who are actively interacting with clients. One purpose of putting our method of online counseling on paper is to enable others to build on our experience and to change it for the better. Future studies based upon our model of online counseling can also shed further light on the efficaciousness of online treatment. In particular, successful replications and generalizations of the findings presented here can address concerns that mental health treatment cannot be provided online in a safe, secure and confidential manner. In addition, future successes with online counseling can begin to change clinical attitudes regarding the potential negative impact of online media on the development of the therapeutic relationship, as well as address concerns that online media inhibits the development of meaningful relationships among clients for helping clinicians take care of patients by email or online.

Investigators are beginning to show success with online counseling. But because their protocols of care are not shared, it is difficult to understand what are the active components of their interventions. In this paper we have presented one protocol of care that has been helpful to our research. Our hope is that other investigators may improve on this protocol. Many clinicians are intrigued with online treatment but do now know how to start such efforts. This protocol provides suggestions

## Competing interests

Dr. Alemi is a consultant to Social Solutions Inc. and Dr. Nemes is the CEO of Social Solutions Inc, a company that is trying to develop an online substance abuse treatment program for patients on Buprenorphine.

## Authors' contributions

FA and MH were the co-principle investigators on the grant from Robert Wood Johnson Foundation and were involved in the conceptualization and implementation of the study, as well as the manuscript composition. SN was the lead psychologist who monitored patients' reactions to the therapeutic intervention. RA was the online counselor for the participants receiving online treatment for substance abuse. JS participated in critical revision of the manuscript for relevance to clinical policy and to address issues regarding the safety and confidentiality of the use of email in healthcare services delivery. DN was involved in discussing the concepts of the improvement teams and in drafting the manuscript. All authors read and approved the final manuscript.

## Appendix

### Case of Toucan

Toucan is the alias of a 47-year-old White female that identifies herself as Catholic. She completed 16 years of education and has a business degree; the longest full time job she had lasted for 5 years. Toucan currently works full time as a waitress. She is not taking medication at this time, has not been in a controlled environment for the past 30 days and was hospitalized for physical problems 14 years ago. One other person depends on her for food, shelter etc.

Toucan has been treated for alcohol abuse 6 times (4 were for detoxification). She has no history of criminal behavior and an unremarkable family history of alcohol, drug and/or psychiatric problems. Last year, Toucan married (these interactions are based on the time period right before the marriage). She reports that she is satisfied with her marriage. Her husband is also an alcoholic. In the past 3 years, she has been living with her son. She spends most of her time with her family. She has two close friends. She reports long lasting personal relationships with her mother, brother and sisters, children and friends, but not with her father. She notes that individuals from this group did abuse her emotionally and physically, but not sexually. She has no history of previous psychiatric problems other than substance abuse.

#### Stage one: establish contact

During the first 2 weeks after Toucan had agreed to participate in this project, her home computer had been installed and communication between Toucan and her substance abuse counselor was evolving. The process took about 2 weeks as both individuals worked out the problems that are inherent to technology. Below are examples of emails sent back and forth between them in this phase:

**From: **Toucan, **To: **Nita Date: 3/13/2002 3:18:23 PM **Subject: **Reply to the phone call: Hi, I am responding to your phone call. I really have no idea, what I am doing. I might be in need of your help. I tried to log on chat line a few times, but it was nobody there to chat with. Thanks for your help, Toucan.

A key problem in starting a chat room is that early clients find it empty and few actively participate in it.

**From: **Toucan, **To: **Nita Date: 3/29/2002 8:49:59 AM **Subject: **testing: Hi, I am trying to send a message. I do not know if it will go through. Toucan.

**From: **Toucan, **To: **Nita Date: 3/29/2002 6:13:42 PM **Subject: **testing: Hi, I am trying again to get through. Toucan.

**From: **Nita, **To: **Toucan Date: 4/1/2002 10:40:28 AM **Subject: **It wasn't your fault: Hi Toucan, This is Nita (your counselor), after speaking with you on the phone, we investigated the situation. There was something wrong with the system and the emails were not reaching my mail box. So you can contact me now. It is important that we maintain good communication. If for some reason you need to reach me here is my number (XXX) XXX-XXXX.

We find it helpful to provide the counselor's phone number to clients in case they need to contact the counselor and cannot do so by email.

**From: **Nita, **To: **Toucan Date: 4/4/2002 7:24:46 AM **Subject: **Consistent contact: Hi Toucan, This is Nita. I emailed you on Monday to let you know that the system is now ready for you to use. To date you have not responded to my email. It is important that we maintain consistent communication so that this process works.

The above emails show a common problem of missed communications. The client started on the system and expected quick responses. An error in the data entry was sending client's emails to a different clinician who was not assigned to Toucan. By the time the problem was identified and the counselor attempted to send a response, the client had abandoned her efforts. Eventually, however, through a few more emails and phone calls the client and the counselor were able to connect. Sometimes missing communications are indicative of relapse, as in the following cases where long periods of time passed without contact:

**From: **Nita, **To: **Toucan Date: 4/14/2002 6:57:52 AM **Subject: **No contact: Hi Toucan, this is Nita. When you and I spoke you informed me that you were comfortable with using the system and had sent me a few emails. That was about three or four weeks ago. Since that time I have not received any emails from you. If you are having problems with your system please call me at this number XXX-XXX-XXXX. There is really no excuse for not keeping in contact with your counselor. The purpose of installing the computer in your home was to make it simple for you to reach your counselor.

**From: **Nita, **To: **Toucan Date: 4/17/2002 8:31:53 AM **Subject: **What's going on?: Hi Toucan, this is Nita. I have not heard from you in at least one month. I have attempted to contact you by email and by phone but have received no responses from you. Is there a problem? I spoke with a gentleman on the phone the Tuesday 4/16, who informed me that you were not available. Here is my number again, XXX-XXX-XXXX.

From time to time, problems of missed emails keep coming back when one party, for various reasons, is not available:

**From: **Toucan, **To: **Nita Date: 6/4/2002 10:51:43 AM **Subject: **Are you on vacation?: Hi Nita, I don't know if you received my message or if you are on vacation. My husband told me you called last night and asked about me. I didn't receive any email from you for about a week. Let me know what is going on. Love, Toucan. PS: If you are on vacation, enjoy! You can also drop by to visit me.

#### Stages two and three: assessment; identify consequences of substance use

The following emails show a number of interactions in which the counselor helps the client explore why she may be ambivalent about change and what are the possible consequences of not changing:

**From: **Nita, **To: **Toucan Date: 4/26/2002 12:35:21 PM **Subject: **Why do you use?: Hey Toucan, this is Nita. You seem to recognize a lot of things. And you also seem to have a lot of answers. Now let's start to address what's going on. I need you to continue to be open and honest. I want to ask you what you would consider are some of the good things about using? I mean what do you get from it? Would you reply and make a list of how and when you feel good about using? I will let you know what day I am available next week so that we can meet. In the mean time let's keep moving forward.

**From: **Toucan, **To: **Nita Date: 4/26/2002 8:36:56 PM **Subject: **Reply to: Why do you use?: Hi Nita, I'm shy by nature. Alcohol makes me more open and free. I drink sometimes when I'm depressed even though I know, alcohol is depressant and I'll be more depressed later. Sometimes it's just for fun. But it is no fun anymore. I guess I was still in denial. I was reading Twenty-Four Hours a Day book today April 26, and my answer is right there. I must go and never stop going to AA. Stop submitting myself to liquor, instead submit myself to a Power greater which I call God. I believe I've done that this time. I'm hurting so bad inside, for what I've done, for my relapse. And I know it has happened only because I'm stubborn, too self-confident, I wanted to stay sober without AA. I thought, I could do it without the help of others. Love, Toucan.

**From: **Toucan, **To: **Nita Date: 4/27/2002 5:24:14 PM **Subject: **Anxiety Hi Nita, I have so much anxiety, it's driving me crazy. I do not know how to deal with it. Can you help? I can't sleep, I can't eat, I can't stay still. Love Toucan.

The above email seems to have been missed by the counselor. There is no sign of further exploration of the sources of anxiety. The counselor, however, returns quickly to the exploration of clients' ambivalence about alcohol use.

**From: **Nita, **To: **Toucan Date: 4/29/2002 10:05:18 AM **Subject: **I want to be sure I understand: Hi. Well first I have to apologize for not responding immediately. I was very sick this weekend, this is the first day that I have been able to get up. This is Nita. You are saying that the good things for you are that alcohol makes you feel free, maybe less inhibited. And then sometimes you drink because you are depressed. Is there anything else? As far as the relapse is concerned, it's important that you move on. Don't beat yourself in the head about it, realize and acknowledge the circumstances that put you in that mode and work at not allowing it to happen again. One important thing you seem to acknowledge is that you can't do it alone. Do you have a sponsor at AA, are you attending regular meetings, are there friends or meeting members that you can socialize and communicate with? Some of the anxiety could be because you are not using your time wisely. An idol mind is dangerous. You said that you are not working any more, so what do you do during the day? Tell me about you. Education, professional skills, family and hobbies. I look forward to hearing from you. Read this email carefully and don't continue to hold a one woman committee meeting in your head.

**From: **Toucan, **To: **Nita Date: 4/29/2002 8:21:44 PM **Subject: **Reply to: I want to be sure I understand: Hi Nita, yes, I started to go to AA again. And I do not have sponsor yet, even though I know I should by now. During the first few days after I came from detox I cleaned my apartment, and did a lot of laundry. I did not do anything during my binge. So this place looked like pigs lived here. Also I was afraid to go outside. Panic attacks and anxiety just took over me. I spent most of my time on phone if I needed to talk to somebody. Now I'm looking for some job. I have a lot of debt which I have to pay off, otherwise I will have to call for bankruptcy, which I don't want to do. Those feelings I did have when I drank that is hopefully in the past. But I need some therapy and time to heal. Yes, sometime I feel as if I have to have a drink, to calm me down, but I know I can't, I know my problems will be worse. Love, Toucan.

**From: **Toucan, **To: **Nita Date: 4/30/2002 8:08:18 PM **Subject: **Reply to: Consequences of use: Hi Nita, I know, I caused all my problems, and the anxiety is a result of them. I had to take care of important things, like charity care. I was in a few hospitals during my binge and have no medical insurance. I have no means to pay for a hospital by myself. I check the papers for ads, call, and go for interviews. I was in a few places to apply. We go to the AA at night. And I talk to people before and after the meetings. I was told long time ago, when I first joined AA, not to ask people for job, it should be recovery only. It would be different if I new somebody well. I told to a few friends I still have that I'm looking for job, and they are trying to help as well. The situation is not very good with jobs now. Everybody is downsizing. I'm dealing with my anxiety as good as I can. Don't be concerned. I will not drink over it. I'll call you before I pick up a drink. Besides my memory of drinking is to fresh and too painful for me to pick up a drink now. Sorry for my English, I'm a foreigner. Love, Toucan.

**From: **Toucan, **To: **Nita Date: 5/22/2002 8:51:39 AM **Subject: **Reply to: Consequences of use: Hi Nita, even though I'm working all this hours, I'm barely able to pay my bills. So money is my big concern. Then my son lives with my sister and I want him back. I know she takes good care of him but I know he needs me, and wants to be with us as well. I know I can't do anything about it and it's killing me. He is a victim of my drinking, he suffers, and I don't know how to ease his pain. It's going to take a time to put my life back to normal, but how do you explain that to a child? He sees his mother sober, and doesn't understand why he cannot come home.

**From: **Toucan, **To: **Nita Date: 5/22/2002 10:33:54 PM **Subject: **Reply to: I see how this concerns you: Hi Nita, If a friend would be telling me about the same problems, my advise would be to take it easy. You can't fix everything overnight. Do what ever you can, have faith, things will get better in time. Nita, I know it will take time, but my son doesn't. He is still a kid who wants to be with his mommy. I live my life today on daily basis. I don't even think about tomorrow. I would get crazy if I did. And yes I have faith. I don't think God saved me, to let me down now. Love, Toucan

**From: **Toucan, **To: **Nita Date: 5/24/2002 8:28:13 AM **Subject: **Reply to: Future focus: Hi Nita, I spend as much time with my son as I can, I'm trying to make the best of the situation. He did have a counseling in the past so I really don't know what more I can do for him. I do not have concerns about the future. I don't think that far. I believe, in time I'll be O.K. My life will be better. I'm very stubborn by nature, and I get what I want if I put my mind into it. Besides nothing can be worst than drinking your life away! And I can see it in AA. Everybody got better, why shouldn't I? Love, Toucan

**From: **Nita, **To: **Toucan Date: 5/28/2002 9:34:28 AM **Subject: **Let's be sure: Hey Toucan, It's Nita. This is a summary of what we have discussed so far. Let me see if I have a clear understanding of our discussion so far. You have several concerns. On one hand you are concerned about getting out of debt and getting your son back as well as staying sober. As far as the future goes, you feel that you don't even think in the future you are trying to live in the day and that by doing all of the things that keep you sober you will have your son with you again. Now, I have request. I want you to write out a message for me about all the things that happens to you that you do not like when you continue to drink. In a month or so from now, I will send the message back to you so that you can see the progress that you are making. As you go through recovery it is important that you remember the reasons you have decided to make changes. So why don't you reply to this message and tell me in as much detail as possible how drinking creates problems for you.

**From: **Toucan, **To: **Nita Date: 5/28/2002 1:33:57 PM **Subject: **Reply to: Let's be sure: Hi Nita, I responded to your request in previous letter, but I can do it again. When I drink, I hate myself, because I don't only create problems for myself, but for people I love. I loose jobs, financial security, I'm depressed, I don't clean, cook, I simply don't care. And that's not me. When I'm sober I'm completely different person. Caring and loving. I know If I continue drinking I will loose even that little I have left. Maybe even my life. I know one thing for sure, I do not want to go back to that pain, loneliness and misery ever again. I deserve better and so do my love ones. Love, Toucan.

#### Stage four: develop a plan for recovery

**From: **Nita, **To: **Toucan Date: 6/5/2002 9:59:05 AM **Subject: **Agreement on need and Individual action: Hi Toucan, This is Nita. In the past weeks, we have been talking about the consequences of your using. You have identified some of the negative consequences that you are concerned about. Let's move on and see if we can come up with a specific plan that can help you change behavior. I have mailed to your home address a brochure that I need you to sign and return. It is called "Contract for Change" and it highlights the activities that you should complete to help change your behavior. I should tell you that you can succeed if that is what you want to do. In the end, it all depends on what you want to accomplish. I cannot decide for you. I can only act as a coach and cheer you on when you succeed and continue to provide you with information that can help to prevent relapse. I will be back in touch shortly.

There is no sign of progression from a resolve to change to actual detailed steps for change as of yet. The counselor will in follow up messages explore how the client plans to change.

#### Stages five and six: admit substance use and mobilize support for change; identify problematic interpersonal relationships

To date, there is no sign of a careful exploration of friends and their contribution to Toucan's drinking habit. But as the emails have shown, her husband is alcoholic and she is increasingly aware of how interacting with another who abuses substances on a regular basis is counterproductive to recovery. Toucan's care is continuing and the counselor is planning to discuss this issue.

#### Stages seven, eight and nine: adjust daily routines, identify substitute routines and create a sense of spirituality & community through group action

Toucan's care is continuing and the counselor has not yet decided to organize a recovery team, even though she has forwarded to her a "Contract for Change" which is the first step in organizing recovery teams.

#### Stage ten: share success with others

This stage is too early as client is still in the process of recovery.

#### Stage eleven: address cycles of relapse

During the time when Toucan was in online counseling there was one major relapse. For approximately one month Toucan did not reply to her emails. When Toucan responds, she explains why the lack of communication had occurred, the experience and reactions to her relapse.

**From: **Nita, **To: **Toucan Date: 4/17/2002 8:31:53 AM **Subject: **What going on?: Hi Toucan, This is Nita. I have not heard from you in at least one month. I have attempted to contact you by email and by phone but have received no responses from you. Is there a problem? I spoke with a gentleman on the phone the Tuesday 4/16, who informed that you were not available. Here is my number again, XXX-XXX-XXXX.

**From: **Toucan, **To: **Nita Date: 4/23/2002 8:02:50 AM **Subject: **Reply to: It wasn't your fault: Hi Nita, as you know by now I did have a relapse. I would like to meet you in person, I really do not know what is wrong with me. I need help. I thought I knew everything and I ended up harming so many people including myself. I hope to hear from you soon. Love, Toucan.

**From: **Nita,**To: **Toucan Date: 4/24/2002 10:46:45 AM **Subject: **Our discussions: Hello Toucan, First I am so glad to hear from you, let me give you a big HUG!!!! Yes, I remember us talking about your relapse. Now, let's get to dealing with what is going on in your life so that another relapse may be prevented. You can do this. Don't quit now. Let's figure out together what is going on. As soon as I can arrange we will have a face to face, but in the mean time I want us to get busy dealing with all that needs to be addressed. I need for you to tell me about the relapse. Tell me what was going on prior to it during and after. Don't be afraid to be blunt, it's important that we understand the entire process in order to attempt to avoid another relapse. And just as a reminder, our discussions are confidential (between me and you) and will not be reported else where without your permission. Come on, Toucan you can do this. I am here for you.

**From: **Toucan, **To: **Nita Date: 4/24/2002 8:59:49 PM **Subject: **Reply to: Our discussions: Dear Nita, what happened was that not only I fired my sponsor but I stopped to go to meetings completely. Not long after, I thought I could do it by myself. I was doing fine, I did not have any urge to drink I was so self confident. One day, at the beginning of April, me and my husband went to a bakery, it was hot, and I said out of the blue "nice cold beer would be good". A bar was just across the street. We did have two at that bar. It was beginning which ended with both of us drunk at the same night. Binge lasted for 16 days. Took me to 5 hospitals and one detox. My husband is alcoholic as well. He is fine now. He went to same detox after I came back. Love, Toucan.

**From: **Nita, **To: **Toucan Date: 4/25/2002 2:24:45 PM **Subject: **Our discussions: Hi Toucan, This is Nita. Thanks for responding. I wonder if you learned anything. If so what? You seem to be on the right track, seeking help is important. Now I need you to address the following: I want to start by asking you why you are here? People have many reasons for seeking treatment. Some feel forced by others or circumstances to do it even though they think that alcohol is not a real problem for them. Others seek it because they are tired of what alcohol has done to their lives. You obviously made a choice to go through the intake interview. But I would like to hear from you and in your words why you are here. Finally give me an idea of what your schedule is like so that we can schedule a face to face.

Even though the counselor has responded in a positive constructive way, we would have preferred a more detailed understanding of the sequence of events leading to relapse and why it continued for 16 days. Without such exploration, the client may not be able to prevent the relapse next time.

#### Stage twelve: make ammends and offer help to others

The client is still in the process of recovery and has not reached this phase.
